# Heavy Metal Uptake of Lettuce and Ryegrass from Urban Waste Composts

**DOI:** 10.3390/ijerph17082887

**Published:** 2020-04-22

**Authors:** Remigio Paradelo, Antía Villada, María Teresa Barral

**Affiliations:** CRETUS Institute, Departamento de Edafoloxía e Química Agrícola, Universidade de Santiago de Compostela, 15782 Santiago de Compostela, Spain; antia.villada.pillado@xunta.gal (A.V.); mteresa.barral@usc.es (M.T.B.)

**Keywords:** composting, urban waste, plant transference, heavy metals

## Abstract

Interest in using urban waste composts as amendments in urban agriculture is growing nowadays. However, concerns about the potential transference of pollutants present in urban waste to the food chain are very relevant when they are recycled for food or animal feed production. Thus, for the safe use of urban waste composts, it has to be assured that no risk of metal transference to plants from compost exists. In this work, the transference of heavy metals from urban waste composts to plants has been studied in an experiment with lettuce and Italian ryegrass, grown in substrates based on five metal-rich composts and a manure vermicompost (included for comparison). A two-month pot experiment was performed under controlled light and temperature conditions, and plant growth and uptake of Cu, Pb, Cd and Zn were analyzed. For both species, the concentration of metals in plant tissue followed the sequence Zn > Cu >> Pb ≈ Cd, the same as the metal concentrations in four out of the five composts. Plant concentrations of Zn, Cu and Cd increased with their concentrations in compost, whereas this relation was not observed for Pb. The ratio between element concentration in plant and compost were much higher for Zn, Cd and Cu than for Pb, showing the lower bioavailability of Pb with respect to other metals.

## 1. Introduction

Urban waste management through composting is gaining attention with the worldwide increase of urbanization. In a moment where the amounts of municipal solid waste are increasing in association to the rise of urban population, composting allows one to manage the large amounts of organic waste produced in urban areas in a simple and efficient way. There are several options for the reutilization of the compost produced from urban wastes, including the amendment of agricultural soils [1,2], production of soilless substrates [3,4], remediation of degraded lands and contaminated soils [5,6] and treatment of polluted wastewaters [7,8]. In addition, the increasing interest in urban agriculture and food production in the urban environment offers new opportunities for the use of urban waste composts [9,10], whether it is performed on soils [11,12] or in soilless media [13,14,15]. In particular, the production of substrates for green roofs and rooftop gardens in cities is an application that demands high amounts of compost [16]. From a wider perspective, composting urban wastes and reusing the compost within the cities will help the advancement of several of the UN Sustainable Development Goals, in particular “Sustainable cities and communities” (goal number 11), “Responsible consumption and production” (goal number 12) and “Climate action” (goal number 13).

While the use of composts has many positive effects, improving soil conditions for plant growth, decreasing erosion risk, increasing biological activity or reducing the mobility of some pollutants [1,6,17], environmental and safety concerns also exist. This is the case specially for composts produced from urban wastes, which may be the source of contaminants such as potentially toxic trace elements, organic pollutants, plastics or pathogenic microorganisms. According to most studies, Cu, Pb and Zn are often the most problematic pollutants in these composts [1,18,19,20,21,22]. These elements are found in urban waste composts, due to their presence in materials such as batteries, paints, electronic, ceramics, printed paper or plastics, as well as their presence in vegetables and other human food materials [18,20]. This concern is particularly relevant when composts are employed for food production, since pollutants can potentially transfer to the food chain through plants and vegetables grown in compost-amended soils or compost-based substrates [18], and also when crops used for animal feed production are involved, in which case pollutants could be transferred indirectly to humans. Thus, for the safe use of compost in urban agriculture and green roof construction, the risk of metal transference from urban waste composts to plants must be assessed. This is even more relevant for the applications of compost in urban environments, that in general involve the use of higher amounts of compost than agricultural applications. This is the case, for example, for green roofs’ soilless substrates, that can be based on high rates of compost, even reaching 100% in some cases [16].

However, studies evaluating the metal uptake of plants growing in soilless substrates with high compost ratios are still scarce and the results obtained in studies with compost-amended soils cannot be directly transposed to this situation. In this context, the objective of this work was to evaluate the possible transference of heavy metals from composts to plants grown in soilless substrates, as well as their relationship with compost composition. For this, we carried out an experiment of plant growth on substrates elaborated with several urban waste composts, all characterized by their high metal contents, using two species: Italian ryegrass and lettuce.

## 2. Materials and Methods

### 2.1. Composts

Four municipal solid waste composts and a manure vermicompost were used in the experiment (Table 1): MSWC1 was obtained by anaerobic fermentation of the biodegradable fraction of municipal solid waste, separated before collection, followed by an aerobic composting step to stabilize the incompletely digested residue. MSWC2 was obtained by composting the source separated organic fraction of municipal solid waste. Both MSWC1 and MSWC2 were provided by industrial composting facilities located in Galicia (Spain). MSGW is a commercial compost obtained from source separated biodegradable municipal solid waste mixed with green waste, and MGSS is compost obtained from municipal garden trimmings mixed with sewage sludge; they both were supplied by an industrial composting facility located in Catalunya (Spain). Finally, a mixed manure vermicompost (MV) was supplied by a local producer in Galicia (Spain); it has been used for comparison purposes. Composted pine bark, obtained through an aerobic transformation in windrows, was supplied by Costiña Orgánica (A Coruña, Spain), and it was employed to prepare the blends with compost used as substrates.

### 2.2. Compost Analysis

For the analysis of the composts, the European methods for the characterization of soil improvers and growing media [23,24,25,26,27] were followed. Briefly, pH was determined in aqueous extracts (substrate/extractant ratio: 1/5 v/v) of fresh samples. Total organic matter (OM) was determined by weight loss on ignition of dried ground samples at 450 °C, and total organic C (TOC) calculated multiplying OM by a factor of 0.58. Total N was measured by Kjeldahl digestion of dried ground samples and steam distillation. Total elements were extracted after the digestion of dried ground sample with HCl and HNO_3_ (3:1 ratio), and the extracts were analyzed using flame atomic absorption spectrometry (Varian SpectraAA 220FS).

### 2.3. Growth Experiment

Five treatments and a control were assayed in the growth experiment, in three replicates each. For this, five substrates were prepared by blending each of the four urban waste composts (MSWC1, MSWC2, MSGW, MGSS) and the manure vermicompost (MV) with the pine bark compost (CPB) at a 50% ratio, on a volume basis. Using these specific composts for substrate production at rates higher than 50% is not possible because of phytotoxicity (as per previous works by Moldes et al. [28] and Barral et al. [3]), and therefore they were not assessed alone. CPB alone was used as a control.

Once the growing media prepared, three pots of 12 cm diameter and 7 cm height were loosely filled with 400 mL of each mixture or the control. Pots were watered and after a short period for surplus water to run off, 50 seeds of Italian ryegrass (*Lolium multiflorum* Lam.) or three seeds of lettuce (*Lactuca sativa* L.) were sown in each pot. A multi-nutrient solution (Welgro Standard Plus commercial; Química Masso S.A., Barcelona, Spain) containing 17% N, 15% K_2_O, 30% P_2_O_5_, 0.13% Fe, 0.052% Mn, 0.06% Zn, 0.02% B and 0.005% Mo, was added initially to the mixtures, in order to obtain final concentrations of 220 mg N L^−1^, 160 mg K L^−1^ and 170 mg P L^−1^ in the substrates. It is important to note that fertilization involves the addition of 0.8 mg Zn L^−1^, a very low quantity in comparison with Zn concentrations in the composts. The pots were covered with a glass plate to reduce evaporative water losses during the first days, and placed in an incubation chamber for 60 days at 20 °C, with a luminous strength of 2150 lux and a 12-h photoperiod.

By the end of the experiment, the number of plants was recorded and they were cut exactly between the root and stalk, and the fresh weight of the shoots was recorded. The plants were then dried at 65 °C and the dry weight was recorded. For the quantification of metal transference to plant, dry plant material was ground and digested with aqua regia (HCl and HNO_3_ in 3:1 ratio) and Cu, Zn, Pb and Cd were analyzed in the extracts by ICP-OES (PerkinElmer, Optima 4300 DV, Waltham, MA, USA).

### 2.4. Statistics

To assess the significance of differences in plant productivity in the greenhouse experiment, one-way ANOVA and Tukey’s multiple range test were performed, using the R statistical package for MacOSX [29] and R commander [30]. Before performing ANOVA, the normality of data and the homogeneity of variance were checked using the Shapiro-Wilk test and the Levene test, respectively.

## 3. Results and Discussion

Table 1 shows a summary of the main properties of the composts. Their pH goes from neutral (MGSS) to alkaline (MSGW), with the exception of pine bark compost (CPB), that was acidic. All the urban waste composts presented high concentrations of heavy metals. Among these, Zn and Cu were the most abundant in all the composts, followed by Pb and Ni, and last, Cd. These concentrations surpassed, in some cases, the levels allowed in the Spanish regulation for the lowest quality organic amendments [31]. According to this law, MSWC1 exceeded the limit for Cd, MSWC2 for Cu, Cd, Pb and Zn, and MGSS for Cu, so they could not be commercialized. Even the composts MV and MSGW, which presented the lowest concentrations of trace elements, would be categorized as class C composts and could not be used in agricultural soils at rates higher than 5 Mg dry matter ha^−1^ year^−1^. More detailed information about phytotoxicity, maturity and heavy metal distribution and availability is available in previous works [22,32,33].

Table 2 shows the results of the growth experiment with ryegrass and lettuce. Despite the metal contents that surpass regulatory thresholds in several composts, in general they improved the productivity of both species with respect to pine bark alone (CPB). This is probably related to the acid pH of the pine bark compost, given that acidity reduces nutrient availability and has a general negative impact on plant growth, as well as to its high C/N ratio that can cause N immobilization. For both species, the yields in the substrates based on the composts MSWC1 and MSWC2 were lower than the other composts. This is likely due to a negative effect of the high salinity of these two composts, as shown by their electrical conductivity values (Table 1), that exceeded the value of 1.5 dS m^−1^, which is usually regarded as inadequate for the agricultural use of compost [34]. In ryegrass, very low germination percentages (under 50%) were observed in those two composts, probably also because of phytotoxicity problems. Since the mean weight for each plant is similar to those grown on other composts, the lower yields must be due to this low germination percentage. For lettuce, lower germination percentages were also observed in MSWC1 and MSWC2, but the lower yields are also due to worse plant development, as shown by the lower average weight per plant.

**Figure 1:**
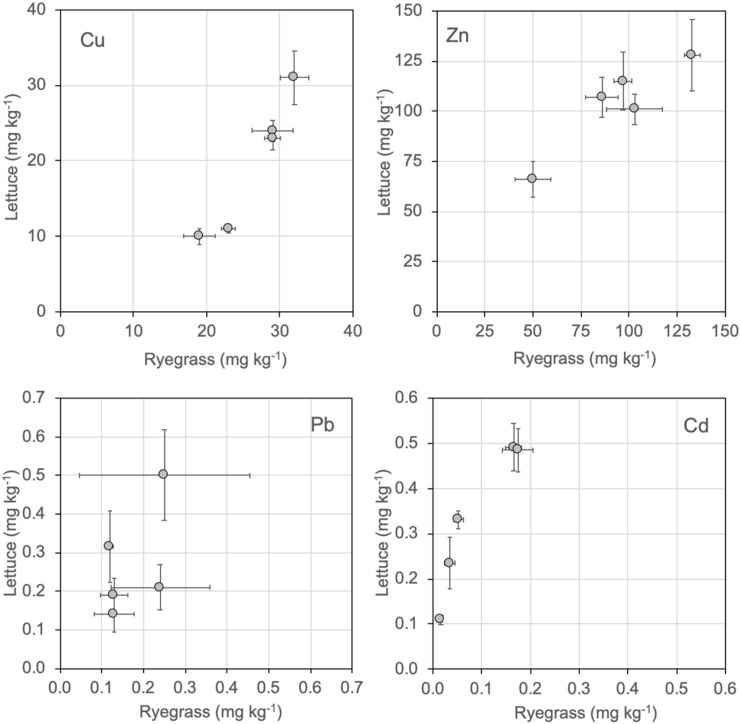
Comparison of metal concentrations (dry weight) found in plants of the two species.

Table 3 and Figure 1 show the concentrations of Cu, Pb, Zn and Cd in lettuce and ryegrass tissue. Whereas Cu, Pb and Zn were present in very similar concentrations in ryegrass and lettuce, transference of Cd was higher to lettuce than to ryegrass. In both species, metal concentrations followed the sequence Zn > Cu >> Pb ≥ Cd, which is the same sequence for metal concentrations in four out of the five composts, and is the sequence commonly observed for metals in plant tissue of different species grown in non-contaminated soils [35], or in organic substrates with compost [36,37,38,39,40]. Concentrations for Cu and Zn are higher than the typical metal contents in plants grown in non-contaminated soils, but overall comparable to values found in plants grown in urban waste-composts [36,37,39]. Cadmium and Pb contents were very low and did not surpass the thresholds established in the EU regulations for leafy vegetables: 0.3 mg kg^−1^ (wet weight) for Pb and 0.20 mg kg^−1^ (wet weight) for Cd [41].

**Figure 2:**
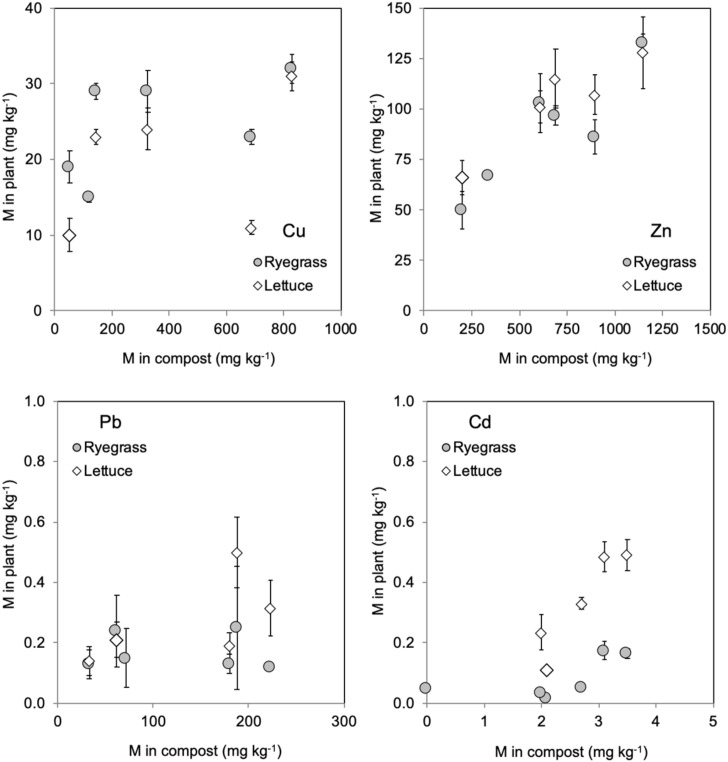
Relationships of concentrations of metals in plants and composts.

Plant concentrations of Zn, Cu and Cd increased with their concentrations in compost, whereas this relation was not observed for Pb (Figure 2), that, as mentioned earlier, was transferred to a much lower extent than the other elements. In this sense, plants grown in the compost MSGW presented the lowest metal concentrations, whereas the plants grown in MSWC2 and MSWC1, especially the first, always presented the highest concentrations. The large differences in metal contents between these composts (Table 1) must be the main factor explaining the results. In addition, the compost MSGW presented a pH which was almost one unit higher than all the other composts, which can also produce a reduction in metal transference. The ratios between the concentration of the element in plant and in compost (transfer factor, averaged for all treatments) were markedly lower for Pb (0.002 in ryegrass and 0.003 in lettuce) than for Cu (0.15 in ryegrass and 0.10 in lettuce), Cd (around 0.05 in ryegrass and 0.15 in lettuce) or Zn (0.15 in ryegrass and 0.18 in lettuce), indicating the lower bioavailability of Pb in compost with respect to the other elements.

This low risk of transference of Pb to plant, despite its high total concentration in some composts, is in agreement with the literature, that shows that this element has exceptionally low mobility and bioavailability in compost-amended soils [20] and low extractability in compost [22,42,43]. The few studies where plants have been grown directly in compost give the same results [37,38,40]. This is an effect of the low mobility of Pb at alkaline pH and its strong affinity for organic matter, that can immobilize it in poorly soluble forms, thus limiting uptake and translocation to plant foliage in harmful amounts. Therefore, the soil-plant barrier protects the food chain in this case and uptake of this element by edible plants should not be a problem for human health [35].

Plant transference of Cu and Zn was higher than for Pb. The high bioavailability of compost Zn and the lower transference of Cu with respect to Zn have also been consistently observed in compost-amended soils [20] and in vegetables growing in different composts [36,37,38,39]. In previous works where we assessed the potential bioavailability of metals in these composts using chemical extractions, we also observed that Zn was the element with the highest potential mobility, either in the composts [22] or in soils amended with them [28]. The lower bioavailability of Cu is attributed to a stronger association of Cu to soil components, so plants are able to regulate its absorption more effectively than in the case of Zn [35]. In any case, these elements do not commonly represent a risk for human health, as phytotoxicity may limit their plant contents in safe levels for chronic ingestion.

Plant Cd in this study remained in concentrations typical of non-contaminated soils. Although Cd concentrations in the plant were low, this element was transferred in similar ratios to Cu or Zn, with respect to its concentration in compost, so that the risk associated to Cd would be higher than for Pb. In addition, the strong influence of pH on the mobility and plant uptake of this element must be kept in mind: for example, Eklind et al. [36] observed a strong negative correlation between substrate pH and Cd concentrations in plants, that increased exponentially with decreasing pH. This resulted in unacceptably high Cd levels in transplants grown in very acid substrates. In this sense, it is important to remember that the alkaline pH of urban wastes composts helps reduce mobility, not only for Cd, but for other metals too. Overall, very low transfers of heavy metals to plant tissues occur in high pH and calcareous soils [20], and it has been observed that, in some cases, pH can be more important in controlling plant availability than actual heavy metal concentrations in compost [36]. In this sense, it is interesting to highlight the fact that, when we compare actual plant transference with measures of metal availability in the composts (Table 4), total concentrations are the best correlated to plant contents, whereas water-extractable fractions are the worst.

The interpretation of the results of this work may differ depending on whether compost is going to be used as a soil amendment or as a component of high-compost substrates. In this study, plants were not grown in soil, but in mixtures with high amounts of urban waste composts, much higher than what is usual in compost-amended soils. Thus, the risk associated to heavy metal uptake by plants in soil would be even lower than what we have found here. In a compost-amended soil, roots quickly grow through the tilled soil depth, where the applied metals accumulate, and then are much less likely to be harmed by the metals [18]. In turn, in pot studies, roots are constrained to the compost, and it has been observed that metal concentrations are higher in plants grown in pots than in the same plants grown in the field [18]. Therefore, the results of this work are more significant for applications where high amounts of compost are used, such as soilless substrates for horticulture or rooftop gardens. Furthermore, the intended use of the substrate is decisive for risk consideration: is it going to be used for food production or for some other use? Obviously, the accumulation of heavy metals is a much less serious problem in container culture of ornamentals than where composts are used in crops for human or animal consumption. And finally, it is essential keeping in mind the influence of pH on metal bioavailability. Since acidity increases metal uptake, the pH of composts and substrates must be controlled carefully in applications involving food production.

## 4. Conclusions

Growing lettuce and ryegrass on substrates based on urban wastes compost has shown that metal uptake in both species followed, in all cases, the sequence Zn > Cu >> Pb ≥ Cd, which is the same sequence as the total concentrations in four out of the five composts. Plant concentrations of Zn, Cu and Cd increased with their concentrations in compost, whereas this relation was not observed for Pb. The ratios between element concentration in plant and compost were much higher for Zn, Cd and Cu than for Pb, showing the lower bioavailability of Pb with respect to other metals. The transference of Cu, Pb and Zn to plant followed similar trends in both species, but Cd was more transferred to lettuce than to ryegrass. We found higher Cu and Zn contents in plants to that which is referenced in the literature as normal for plants grown in non-contaminated soils, and similar or lower for Pb and Cd, and, in any case, far from the EU thresholds for food security protection. In summary, the risk of plant transference of heavy metals in amounts that represent a concern to the food chain is low, even when growing vegetables directly on compost with high concentrations of these elements.

## Figures and Tables

**Table 1:** Properties of the composts used in substrate formulations. MSWC1 and MSWC2: composts obtained from source separated organic fraction of municipal solid waste; MSGW: compost obtained from source separated organic fraction of municipal solid waste mixed with green waste; MGSS: compost obtained from municipal garden trimmings mixed with sewage sludge; MV: manure vermicompost; CPB: composted pine bark; EC: electrical conductivity; OM: total organic matter; nd: under the detection limit; (‡): not allowed for marketing according to maximum trace element concentrations published in the Spanish Fertilizers Law for organic amendments.

	Bd (Mg m^−3^)	pH	EC (dS m^−1^)	OM (g kg^−1^)	Total N (g kg^−1^)	C/N	Total Cu (mg kg^−1^)	Total Zn (mg kg^−1^)	Total Pb (mg kg^−1^)	Total Cd (mg kg^−1^)	Class according to Spanish Fertilizers Law
MSWC1	0.42	8.4	2.3	490	17	17	325	608	188	3.5	(‡)
MSWC2	0.53	8.2	2.4	397	15	15	829	1149	223	3.1	(‡)
MSGW	0.53	9.2	1.2	429	17	14	52	200	62	2.1	C
MGSS	0.47	7.3	1.4	515	18	15	688	896	180	2.7	(‡)
MV	0.59	7.9	0.7	376	10	21	144	689	33	2.0	C
CPB	0.45	5.3	0.4	914	2.8	194	120	340	72	nd	C
Heavy metal limits		Spanish Fertilizers Law					400	1000	200	3	
		EPA Class A Biosolids					1500	2800	300	39	

**Table 2:** Germination and shoot weight of the plants grown in the mixtures of composts with pine bark compost. Different letters in each column mean statistically significant differences in the Tukey test at *p* < 0.05. MSWC1 and MSWC2: composts obtained from source separated organic fraction of municipal solid waste; MSGW: compost obtained from source separated organic fraction of municipal solid waste mixed with green waste; MGSS: compost obtained from municipal garden trimmings mixed with sewage sludge; MV: manure vermicompost; CPB: composted pine bark.

Ryegrass	Number of Plants	Fresh Weight (g)	Dry Weight (g)	Dry Weight/Plant
MSWC1	20 ± 3 a	8.4 ± 2.5 a	1.0 ± 0.3 ab	0.05
MSWC2	24 ± 6 ab	9.7 ± 2.8 a	1.0 ± 0.3 ab	0.04
MSGW	43 ± 6 c	9.9 ± 1.8 a	1.4 ± 0.03 bc	0.03
MGSS	38 ± 3 c	17.0 ± 2.5 b	1.8 ± 0.2 c	0.05
MV	36 ± 5 bc	16.9 ± 2.4 b	1.8 ± 0.3 c	0.05
CPB	24 ± 5 ab	5.5 ± 2.3 a	0.6 ± 0.2 a	0.03
**Lettuce**	**Number of Plants**	**Fresh Weight (g)**	**Dry Weight (g)**	**Dry Weight/Plant**
MSWC1	2.3 ± 1.2 a	3.8 ± 1.4 a	0.19 ± 0.1 a	0.08
MSWC2	2 ± 0 a	5.7 ± 0.9 a	0.26 ± 0.04 ab	0.13
MSGW	3 ± 0 a	22.6 ± 2.4 c	1.1 ± 0.3 d	0.37
MGSS	3 ± 0 a	13.4 ± 0.4 b	0.57 ± 0.04 bc	0.19
MV	3 ± 0 a	19.5 ± 1.5 c	0.88 ± 0.1 cd	0.29
CPB	2.7 ± 0.3 a	2.4 ± 0.7 a	0.08 ± 0.01 a	0.03

**Table 3:** Heavy metal concentrations (mg kg^−1^, dry weight) in plants grown in the mixtures of composts with pine bark compost. Composition of lettuce grown in CPB is not shown because the amount of sample was insufficient for analysis. Different letters denote significant differences between composts in the Tukey test at *p* < 0.05. MSWC1 and MSWC2: composts obtained from source separated organic fraction of municipal solid waste; MSGW: compost obtained from source separated organic fraction of municipal solid waste mixed with green waste; MGSS: compost obtained from municipal garden trimmings mixed with sewage sludge; MV: manure vermicompost; CPB: composted pine bark.

Ryegrass	Cu	Zn	Pb	Cd
MSWC1	29 ± 3 c	103 ± 14 c	0.3 ± 0.2 a	0.17 ± 0.02 b
MSWC2	32 ± 2 c	133 ± 4 d	0.12 ± 0.01 a	0.17 ± 0.03 b
MSGW	19 ± 2 ab	50 ± 9 a	0.2 ± 0.1 a	0.02 ± 0.004 a
MGSS	23 ± 1 b	86 ± 8 bc	0.13 ± 0.03 a	0.05 ± 0.01 a
MV	29 ± 1 c	97 ± 5 c	0.13 ± 0.05 a	0.03 ± 0.01 a
CPB	15 ± 0.6 a	67 ± 1 ab	0.10 ± 0.1 a	0.05 ± 0.004 a
Reference values [35]	5–10	25–47	0.4–5	0.1–0.6
**Lettuce**	**Cu**	**Zn**	**Pb**	**Cd**
MSWC1	24 ± 1 b	101 ± 8 b	0.50 ± 0.12 b	0.49 ± 0.05 c
MSWC2	31 ± 3 c	128 ± 18 b	0.32 ± 0.09 ab	0.48 ± 0.05 c
MSGW	10 ± 1 a	66 ± 9 a	0.21 ± 0.06 a	0.11 ± 0.01 a
MGSS	11 ± 1 a	107 ±10 b	0.19 ± 0.04 a	0.33 ± 0.02 b
MV	23 ± 2 b	115 ± 14 b	0.14 ± 0.05 a	0.23 ± 0.06 b
Reference values [35]	6–8	44–73	0.7–3.6	0.40–0.66

**Table 4:** Correlations between heavy metal concentrations in plants grown in the mixtures of composts and pine bark compost and the results of chemical extractions of composts from Paradelo et al. [22] for all elements together. Significance of correlations is indicated as follows: ** significant at a *p*-value of 0.01; *** significant at a *p*-value of 0.001.

	Total	DTPA-Extractable	TCLP	Water-Extractable
Ryegrass	0.83 ***	0.74 ***	0.78 ***	0.28
Lettuce	0.80 ***	0.76 **	0.70 **	0.22

## References

[1] Hargreaves J.C., Adl M.S., Warman P.R. (2008). A review of the use of composted municipal solid waste in agriculture. Agric. Ecosys. Environ..

[2] Srivastava V., de Araujo A.S.F., Vaish B., Bartelt-Hunt S., Singh P., Singh R.P. (2016). Biological response of using municipal solid waste compost in agriculture as fertilizer supplement. Rev. Environ. Sci. Biotechnol..

[3] Barral M.T., Moldes A.B., Cendón Y., Díaz-Fierros F. (2007). Assessment of municipal solid waste compost quality using standardized methods before preparation of plant growth media. Waste Manag. Res..

[4] Herrera F., Castillo J.E., Chica A.F., López Bellido L. (2008). Use of municipal solid waste compost (MSWC) as a growing medium in the nursery production of tomato plants. Bioresour. Technol..

[5] Farrell M., Jones D.L. (2009). Critical evaluation of municipal solid waste composting and potential compost markets. Bioresour. Technol..

[6] Park J.H., Lamb D., Paneerselvam P., Choppala G., Bolan N., Chung J.-W. (2011). Role of organic amendments on enhanced bioremediation of heavy metal (loid) contaminated soils. J. Hazard. Mater..

[7] Silvetti M., Demurtas D., Garau G., Deiana S., Castaldi P. (2017). Sorption of Pb, Cu, Cd, and Zn by municipal solid waste composts: Metal retention and desorption mechanisms. Clean Soil Air Water.

[8] Paradelo R., Vecino X., Moldes A.B., Barral M.T. (2019). Potential use of composts and vermicomposts as low-cost adsorbents for dye removal: An overlooked application. Environ. Sci. Pollut. Res..

[9] Cogger C.G. (2005). Potential compost benefits for restoration of soils disturbed by urban development. Compost Sci. Util..

[10] Heyman H., Bassuk N., Bonhotal J., Walter T. (2019). Compost quality recommendations for remediating urban soils. Int. J. Environ. Res. Public Health.

[11] Sotamenou J., Parrot L. (2013). Sustainable urban agriculture and the adoption of composts in Cameroon. Int. J. Agric. Sustain..

[12] Ulm F., Avelar D., Hobson P., Penha-Lopes G., Dias T., Máguas C., Cruz C. (2019). Sustainable urban agriculture using compost and an open-pollinated maize variety. J. Clean. Prod..

[13] Grard B.J.-P., Bel N., Marchal N., Madre F., Castell J.-F., Cambier P., Houot S., Manouchehri N., Besancon S., Michel J.-C. (2015). Recycling urban waste as possible use for rooftop vegetable garden. Future Food J. Food. Agric. Soc..

[14] Matlock J.M., Rowe D.B. (2017). Does compost selection impact green roof substrate performance? Measuring physical properties, plant development, and runoff water quality. Compost Sci. Util..

[15] Paradelo R., Basanta R., Barral M.T. (2019). Water-holding capacity and plant growth in compost-based substrates modified with polyacrylamide, guar gum or bentonite. Sci. Hortic. Amst..

[16] Eksi M., Rowe D.B., Fernández-Cañero R., Cregg B.M. (2015). Effect of substrate compost percentage on green roof vegetable production. Urban For. Urban Green..

[17] Diacono M., Montemurro F. (2010). Long-term effects of organic amendments on soil fertility. A review. Agron. Sustain. Dev..

[18] Chaney R.L., Brown S.L., Malik M., Siebielec G., Kukier U., Ryan J.A., Angle J.S., Stofella P.J., Kahn B.A. (2001). Heavy metal aspects of compost use. Compost Utilization in Horticultural Cropping Systems.

[19] Tittarelli F., Pettruzelli G., Pezzarossa B., Civilini M., Benedetti A., Sequi P., Díaz L.F., de Bertoldi M., Bidlingmaier W., Stentiford E. (2007). Quality and agronomic use of compost. Compost Science and Technology.

[20] Smith S.R. (2009). A critical review of the bioavailability and impacts of heavy metals in municipal solid waste composts compared to sewage sludge. Environ. Int..

[21] Lopes C., Herva M., Franco-Uría A., Roca E. (2011). Inventory of heavy metal content in organic waste applied as fertilizer in agriculture: Evaluating the risk of transfer into the food chain. Environ. Sci. Pollut. Res..

[22] Paradelo R., Villada A., Devesa-Rey R., Moldes A.B., Domínguez M., Patiño J., Barral M.T. (2011). Distribution and availability of trace elements in municipal solid waste composts. J. Environ. Monitor..

[23] AENOR (Asociación Española de Normalización y Certificación) (2001). Soil Improvers and Growing Media. Determination of Ph.

[24] AENOR (Asociación Española de Normalización y Certificación) (2001). Soil Improvers and Growing Media. Determination of Electrical Conductivity Norma Española UNE-EN 13038.

[25] AENOR (Asociación Española de Normalización y Certificación) (2001). Soil Improvers and Growing Media—Determination of Organic Matter Content and Ash.

[26] AENOR (Asociación Española de Normalización y Certificación) (2002). Soil Improvers and Growing Media—Extraction of Aqua Regia Soluble Elements.

[27] AENOR (Asociación Española de Normalización y Certificación) (2002). Soil Improvers and Growing Media—Determination of Nitrogen—Part 1: Modified Kjeldahl Method.

[28] Moldes A., Cendón Y., López E., Barral M.T. (2006). Biological quality of potting media based on MSW composts: A comparative study. Compost Sci. Util..

[29] R Core Team (2018). R: A Language and Environment for Statistical Computing.

[30] Fox J., Bouchet-Valat M. Rcmdr: R Commander. R Package Version 2.6-1. http://socserv.socsci.mcmaster.ca/jfox/Misc/Rcmdr/.

[31] Ministerio de Agricultura (2013). Real Decreto 506/2013, de 28 de junio, sobre productos fertilizantes. B.O.E..

[32] Paradelo R., Moldes A.B., Prieto B., Sandu R.-G., Barral M.T. (2010). Can stability and maturity be evaluated in finished composts from different sources?. Compost Sci. Util..

[33] Paradelo R., Villada A., Barral M.T. (2018). Chemical fractionation of trace elements in a metal-rich amphibolite soil amended with municipal solid waste composts. Waste Biomass Valoriz..

[34] Masaguer A., Benito M., Moreno J., Moral R. (2008). Evaluación de la calidad del compost. Compostaje.

[35] Kabata-Pendias A., Pendias H. (1984). Trace Elements in Soils and Plants.

[36] Eklind Y., Rämert B., Wivstad M. (2001). Evaluation of growing media containing farmyard manure compost, household waste compost or chicken manure for the propagation of lettuce (*Lactuca sativa* L.) transplants. Biol. Agric. Hortic..

[37] Zubillaga M.S., Lavado R.S. (2002). Heavy metal content in lettuce plants grown in biosolids compost. Compost Sci. Util..

[38] Intawongse M., Dean J.R. (2006). Uptake of heavy metals by vegetable plants grown on contaminated soil and their bioavailability in the human gastrointestinal tract. Food Addit. Contam..

[39] Mupondi L.T., Mnkeni P.N.S., Brutsch M.O. (2006). Evaluation of pine bark or pine bark with goat manure or sewage sludge cocomposts as growing media for vegetable seedlings. Compost Sci. Util..

[40] Mininni C., Grassi F., Traversa A., Cocozza C., Parente A., Miano T., Santamaria P. (2015). (*Posidonia oceanica* L.) based compost as substrate for potted basil production. J. Sci. Food Agric..

[41] European Communities Council (2001). Commission Regulation 466/2001 setting maximum levels for certain contaminants in foodstuffs. Off. J. Eur. Commun..

[42] Grimes S.M., Taylor G.H., Cooper J. (1999). The availability and binding of heavy metals in compost derived from household waste. J. Chem. Technol. Biotechnol..

[43] Song Q.J., Greenway G.M. (2004). A study of the elemental leachability and retention capability of compost. J. Environ. Monitor..

